# Thrombotic microangiopathy in patients with sickle cell disease

**DOI:** 10.1590/1984-0462/2024/42/2023108

**Published:** 2024-05-27

**Authors:** Gabriella Biasi Carrasco, Patricia Belintani Blum, Josefina Aparecida Pellegrini Braga

**Affiliations:** a Universidade Federal de São Paulo, São Paulo, SP, Brasil

**Keywords:** Sickle cell disease, Microangiopathic hemolytic anemia, Acute chest syndrome, Doença falciforme, Anemia hemolítica microangiopática, Síndrome torácica aguda

## Abstract

**Objective:**

To describe two cases of patients who had thrombotic microangiopathy (TMA) associated with sickle cell disease (SCD).

**Case description:**

Both patients started with a painful crisis and had acute chest syndrome during hospitalization. They showed significant worsening of hemolytic anemia, with very high levels of lactate dehydrogenase, thrombocytopenia, lowered level of consciousness, organ damage and the presence of schistocytes in peripheral blood. Due to the possibility of TMA, despite the very rare association with SCD, they were treated with fresh frozen plasma replacement and plasmapheresis, with good response.

**Comments:**

TMA is a serious, life-threatening disease, characterized by microangiopathic hemolytic anemia, thrombocytopenia, and organ damage. The association of SCD and TMA is difficult to diagnose, since they can share a similar clinical presentation. Recognizing this association and promptly instituting treatment may impact the survival of these patients.

## RESUMO

**Objetivo:**

Descrever dois casos de pacientes que apresentaram microangiopatia trombótica (MAT) associada à doença falciforme (DF).

**Descrição do caso:**

Ambos os pacientes iniciaram com crise dolorosa e apresentaram síndrome torácica aguda durante a internação. Eles apresentaram piora significativa da anemia hemolítica, com níveis muito elevados de lactato desidrogenase, trombocitopenia, rebaixamento do nível de consciência, lesão de órgãos e presença de esquistócitos no sangue periférico. Diante da possibilidade de MAT, apesar da associação muito rara com DF, eles foram tratados com reposição de plasma fresco congelado e plasmaférese, com boa resposta.

**Comentários:**

A MAT é uma doença grave e com risco de vida, caracterizada por anemia hemolítica microangiopática, trombocitopenia e danos a órgãos. A associação de DF e MAT é de difícil diagnóstico, pois as duas podem ter apresentação clínica semelhante, portanto reconhecer essa associação e instituir o tratamento prontamente pode ter grande impacto na sobrevida desses pacientes.

## INTRODUCTION

Thrombotic microangiopathy (TMA) is a rare situation characterized by the triad: thrombocytopenia, non-immune-mediated microangiopathic hemolytic anemia and target-organ ischemia, in the presence of schistocytes in peripheral blood smears.^1^ This triad occurs due to changes in microcirculation, leading to platelet aggregation, hemolysis by fragmentation of red blood cells and ischemia of the affected site.^1^ TMA can be primary or secondary. Among the primary type, we can highlight thrombotic thrombocytopenic purpura (TTP), which occurs due to acquired or congenital deficiency of the enzyme a disintegrin and metalloprotease with thrombospondin type 1 motif, member 13 (ADAMTS-13); hemolytic uremic syndrome (HUS) mediated by Shiga toxin released by some microorganisms, especially *Escherichia coliO157:H7*; and complement-mediated TMA (atypical HUS), caused by alterations in the inhibition of the complement pathway, which may be congenital or autoimmune.^2^ Among the secondary causes of TMA, we can highlight drug-induced causes, malignant tumors, autoimmunity, infections and transplant.^2^

In Sickle Cell Disease (SCD), the vaso-occlusive crisis occurs, leading to hemolysis and ischemia, with organ damage, which may cause extreme pain, priapism, stroke, and acute chest syndrome (ACS).^3^ In Brazil, it is estimated that there are currently between 60 thousand and 100 thousand patients with SCD.^4^

The association of SCD and TMA is very rare and difficult to diagnose, as both condition cause organ damage and hemolytic anemia, with thrombocytopenia being more common in TMA than in SCD, and in this situation it can occur in splenic sequestration or sepsis.^3^ Therefore, it is essential to consider this possibility in patients with SCD, so that the diagnosis can be made, and plasma therapy instituted.

We will report two pediatric patients with SCD who had TMA.

## CASE REPORT

### Case 1

A nine-year-old afro-descendant boy, with SCD (HbSS), was taken by his mother to the emergency room due to trauma to his left elbow, without fracture. On the same day, he developed a vaso-occlusive crisis, with severe pain in his left upper limb, lumbar region, and lower left limb, requiring opioids. At the first examination, he presented with moderate pallor, no respiratory distress. On the third day of hospitalization, he developed fever, jaundice, worsening pallor, hemoglobinuria, and thrombocytopenia. A hemolytic crisis with probable infection was considered, and red blood cells were transfused and ceftriaxone was introduced. On the seventh day, no longer in pain, he presented severe, sudden respiratory discomfort, with vomiting and alteration of the level of consciousness due to central nervous system bleeding, reduced renal function requiring hemodialysis, worsening of hemolytic anemia, with lactate dehydrogenase (LDH) of 1440 u/L and thrombocytopenia reaching 20,000/mm^3^, with the presence of schistocytes in peripheral blood and normal clot test assays. A TMA diagnostic hypothesis was proposed, and therapy was started with fresh frozen plasma replacing. He received 10 mL/Kg, twice a day, for 15 days and then once a day in the third week (at that time, plasmapheresis was not available in our hospital). ADAMTS-13 was collected, with a result of 58%, although the patient had already received plasma. He had completely recovery of clinical and laboratory conditions, and no recurrence after six years of follow-up (Table 1).

**Table 1:** Case 1 laboratorial data.

	Admission	TMA diagnosis	Discharge	After treatment
Hb	9.0	7.1	8.5	8.9
Plt	120,000	20,000	81,000	358,000
LDH	736	1440	166	659
UCB	1.23	14.2	0.16	-
CB	0.51	6.6	0.72	-
AST	-	112	30	35
ALT	-	10	22	10
U	28	147	154	19
Cr	0.46	2.54	1.08	0.35

TMA: thrombotic microangiopathy; Hb: hemoglobin (g/dL); Plt: platelet (mm^3^); LDH: lactate dehydrogenase (140–280 U/L); UCB: unconjugated bilirubin; CB: conjugated bilirubin; AST: aspartate aminotransferase; ALT: alanine aminotransferase, U: urea, Cr: creatinine.

### Case 2

A 13-year-old afro-descendant boy, with SCD (HbSC). His mother took him to the emergency room because he had been complaining of severe abdominal pain in the left hypochondrium for a day. The boy denied fever and had no other complaints. On physical examination, he presented moderate pallor, without respiratory distress, with a diffusely painful abdomen on palpation, and a spleen 6 cm from the left costal margin. A diagnostic hypothesis of splenic sequestration was proposed, and analgesia and blood transfusion were performed. There was regression of the spleen. He developed fever, respiratory distress, hypoxemia, radiological image compatible with ACS and lowered level of consciousness. Skull tomography was normal. He started showing significant hemolysis with hemoglobinuria, 5607 u/L LDH, 78,000/mm³ thrombocytopenia, with schistocytes in the peripheral blood, normal clot test assays, negative direct Coombs, reduced renal function and worsening of liver enzymes (total bilirubin at 4.71 mg/dL, with direct, 3.47 mg/dL, and indirect, 1.24 mg/dL; aspartate transaminase [AST] 5383 u/L and alanine transaminase [ALT] 1794 u/L). Due to the triad of microangiopathic hemolysis, thrombocytopenia, and organ damage, we proposed the diagnostic hypothesis of TMA (ADAMTS-13 dosage was not available in our hospital at that time), and we started fresh frozen plasma replacing (10 mL/Kg, twice a day) with subsequent plasmapheresis. He had 13 daily plasmapheresis sessions, showing significant improvement after 15 days treatment. Subsequently, there was complete recovery of the clinical and laboratory conditions, with no recurrence after four years of follow-up (Table 2).

**Table 2:** Case 2 laboratorial data.

	Admission	TMA Diagnosis	Discharge	After treatment
Hb	8.8	8.8	9.7	9.8
Plt	187,000	78,000	303,000	409,000
LDH	552	5607	605	324
UCB	0.6	1.24	0.31	0.42
CB	0.48	3.47	0.25	0.27
AST	615	5383	85	28
ALT	209	1794	61	20
U	39	26	28	22
Cr	0.74	0.75	0.41	0.51

TMA: thrombotic microangiopathy; Hb: hemoglobin (g/dL); Plt: platelet (mm^3^); LDH: lactate dehydrogenase (140–280 U/L); UCB: unconjugated bilirubin; CB: conjugated bilirubin; AST: aspartate aminotransferase; ALT: alanine aminotransferase, U: urea, Cr: creatinine.

## DISCUSSION

The diagnostic criteria for TMA are:

1. Microangiopathic hemolytic anemia (hemoglobin <12 g/dL) with a negative direct Coombs test, more than two schistocytes in a microscopic field with a 100 times magnitude, and an increase in LDH above institutional baseline;
2. Thrombocytopenia (platelet count <100,000/ mm^3^);
3. Variable severity of organ dysfunction, with no signs of disseminated intravascular coagulation (DIC).^5^

The two cases described met all these criteria for diagnosing TMA.

TMA can be classified according to its pathophysiology into: ADAMTS-13 deficiency-mediated, as in TTP and thrombocytopenia-associated multiple organ failure (TAMOF); complement-mediated, as in atypical HUS; and Shiga toxin-mediated.^2^

As our patients had been under medical care since the first year of life, without thrombocytopenia, and after treatment they remained with normal platelets for years and without signs of TMA, it is very unlikely that this is congenital PTT, although they did not have ADAMTS-13 dosage after the end of treatment. Immune PTT is very rare in children and usually presents with platelets <30,000; as both cases resolved within a few days, this possibility is also unlikely, although it was also not possible to test for ADAMTS-13 inhibitor. TAMOF is usually associated with sepsis and consumption coagulopathy, and this situation was not present in our patients.^6^ In the case of complement-mediated TMA, there may be less response to plasma therapy and recurrence is frequent.^7^

The association of SCD and TMA is very rare, with few cases in the literature. Little is known whether the two conditions are directly related, and, because they present similar clinical and laboratory aspects, their association represents a diagnostic challenge.

Shome et al. reported a series of ten cases of patients with SCD who had TMA.^8^ All patients with TMA had ACS and, when comparing the group with TMA, with a group with ACS without TMA, they found that patients with TMA had lower platelet and hemoglobin values, higher LDH and changes in renal and hepatic biochemical markers.^8^ Thrombocytopenia combined with LDH above 1000 u/L occurred in all patients with TMA and in no patient in the other group.^8^

Our patients also had ACS, thrombocytopenia and LDH above 1000 u/L, schistocytes, as well as acute renal and hepatic alterations. These observations agree with Shome *et al*. that the triad of thrombocytopenia, schistocytes and LDH above 1000 u/L would be sufficient to suggest TMA in these patients.^8^

Both patients had neurological symptoms with lowered level of consciousness, a symptom also frequently reported by other authors.^8-11^

Regarding the pathophysiology of TMA secondary to SCD, Shome *et al.* hypothesized that the endothelial activation that occurs during a vaso-occlusive crisis may lead to greater release of multimers of von Willebrand factor, and inhibition or reduction of the activity of ADAMTS-13 by hyperhemolysis, proteolysis and/or reduction of its synthesis, thus decreasing the cleavage of von Willebrand multimers, which leads to microangiopathic thrombosis.^8^ This hypothesis can be supported by the fact that these patients respond well to plasma therapy.^8^

Plasma replacement therapy is useful to replace substances that may be missing in the TMA, such as ADAMTS-13 and complement inhibitors. Plasmapheresis, in turn, is useful to remove inhibitors (autoantibodies) present in the plasma. Plasmapheresis resulted in a significant improvement in survival from 10 to 78% in patients with TMA due to TTP, being crucial for a favorable outcome in these conditions.^12^ Regarding TMA associated with SCD, several authors have shown a good response with plasma exchange therapy,^8-11^ as well as our patients. The first of them only received plasma replacement and the second had plasma exchange, both with progressive clinical and laboratory improvement.

As the literature data are just a few retrospective reports, and the factors involved in the pathophysiology of this association are not exactly known, we cannot state that plasma therapy is the best treatment. However, based on the good response of our patients and the literature, plasmapheresis would be indicated daily and maintained until at least the platelet count returns to normal. It is also known that the level of LDH, which reflects both tissue ischemia and hemolysis, is also an important marker of response to treatment.^13^

Our patients had no recurrence after the end of plasma therapy, a fact also verified by other authors, showing that recurrence seems to be rare.^8^ It is worth raising the possibility that SCD could be considered a secondary cause of TMA that responds to plasma therapy.

In conclusion, despite the rarity of the condition, and the little pathophysiological knowledge we have about it, it is essential to consider the occurrence of TMA in all sickle cell patients with significant hemolysis, associated with thrombocytopenia and target-organ damage, mainly kidneys, liver, and CNS. Remembering this diagnosis and promptly instituting treatment can have a great impact on the survival of these patients.
